# Combined use of rubber bands and patient’s own hair to optimize scalp surgical field exposure and secure wound dressings

**DOI:** 10.1016/j.jpra.2025.11.023

**Published:** 2025-11-22

**Authors:** Jin-Yuan Ma, Ya-Di Li, Xi Chen, Yu-Rong Li

**Affiliations:** Department of Dermatology, Beijing Tongren Hospital, Capital Medical University, Beijing 100730, China

**Keywords:** Scalp surgery, Preoperative hair management, Postoperative bandaging, Wound dressing fixation

## Clinical issues

During scalp surgeries in patients with medium to long hair, hair can obstruct the surgical field, increase contamination risks, and become tangled with sutures. These issues reduce surgical efficiency, raise procedural difficulty, and heighten infection risks.^1^

Current hair management methods—tape, gels, clips, or films—often suffer from poor fixation, patient discomfort, or sticky residue.^2^, ^3^, ^4^, ^5^ After surgery, hair tends to stick to wound dressings, causing shift or detach. Movement can create friction, impairing healing or even reopening the wound. Moreover, inconvenient hair management may lead patients to touch the wound area frequently, further increasing infection risks and interfering with recovery.

To address these issues, this study proposes two improved methods:

1. A novel preoperative hair bundling technique designed to securely expose the surgical area during preparation, preventing surrounding hair from obstructing the surgical field.
2. An innovative bandaging method that effectively secures existing non-adhesive dressings on the scalp using patient’s own hair around the surgical site.

## Preoperative hair braiding method

During preoperative preparation, the skin around the lesion is kept intact without shaving. Given the patient's long and thick hair, as well as the easy mobility of hair strands, traditional fixation methods—disposable medical adhesive tape or securing a ponytail with hair clips—prove inadequate for effectively fixing hair around the excision site. While tying a ponytail and employing hair clips could manage most of the hair on one side, short strands remain difficult to secure. Additionally, high overall tension in the patient's scalp may increase procedural difficulty and perioperative risks during surgery.

Thus, an alternative approach is adopted: small hair bundles around the lesion area are identified and separated, and each of these hair bundles is sequentially secured using colored rubber bands. This method ensures that the hair is neatly organized and held away from the lesion site, facilitating unobstructed access during surgical procedures. Distinctively colored rubber bands also aid in the clear identification and management of individual hair bundles, contributing to the overall precision and orderliness of the preparation process while reducing scalp tension.

**Figure 1:**
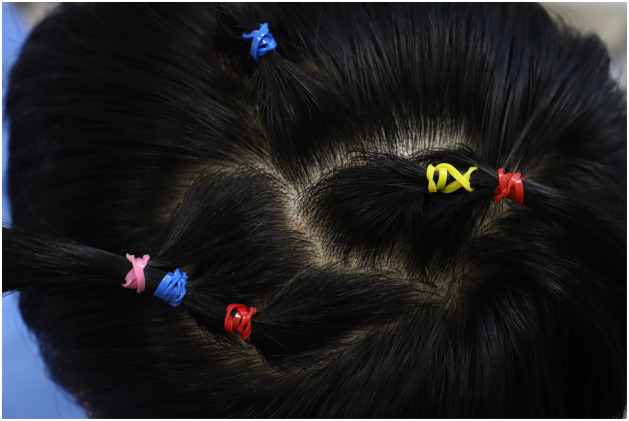
To optimize the surgical field exposure around the lesion, the patient's hair is managed as follows: Firstly, small bundles of hair surrounding the lesion area are identified and separated. Subsequently, each of these hair bundles is sequentially secured using colored rubber bands. This method ensures that the hair is neatly organized and held away from the lesion site, facilitating unobstructed access during surgical procedures. The use of distinctively colored rubber bands also aids in the clear identification and management of individual hair bundles, contributing to the overall precision and orderliness of the preparation process.

To address these challenges, we use rubber bands to secure hair around the lesion, thereby minimizing hair removal in the surgical area. The specific procedures are as follows: First, identify the lesion site. Then, at appropriate positions around it, grasp a small bundle of hair (approximately 100–150 strands) and tie each bundle using colored elastic bands. Continue gathering and securing adjacent hair bundles with elastic bands sequentially around the lesion (Figure 1).

This method establishes a stable demarcation for surgery while minimizing interference from long hair intraoperatively. Even with uneven hair distribution around the lesion, fixation with elastic bands still achieves a stable result. Postoperatively, rubber bands can be simply cut to release hair.

## Postoperative bandaging

**Figure 2:**
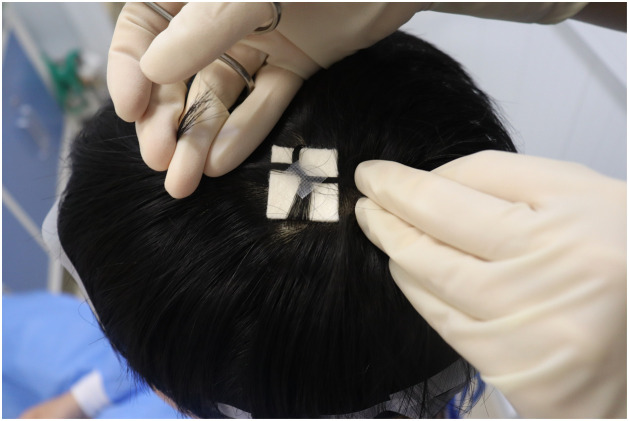
Securing scalp wound dressings utilizing medical adhesive tape and the patient's own hair. After applying a sterile dressing over the wound site, select a small quantity of hair (approximately 20–30 strands each) from 3 o'clock, 6 o'clock, 9 o'clock, and 12 o'clock positions surrounding the patient's surgical area. Then, two bundles of hair from 3 o'clock and 9 o'clock are crossed, and medical adhesive tape is used on the intersection to enhance fixation. To further reinforce the stability of the dressing, bundles of hair from 6 o'clock and 12 o'clock are twisted and fixed using medical adhesive tape as before.

During postoperative bandaging, first cover the incision with a sterile dressing, and select a small quantity of long hair (approximately 20–30 strands each) from the 3, 6, 9, 12 o'clock positions around the surgical area. If the surgical site is close to the hairline with no hair in some surrounding areas, only two of the four directions (e.g., 3 and 9 o'clock) can be selected for combination, and the sterile dressing can still be securely fixed. Gently pull these four hair bundles to the opposite side of the surgical field and cross them over the dressing to form a stable external fixation structure. Subsequently, use medical adhesive tape to secure the hair over the dressing, ensuring the bandage’s firmness and stability (Figure 2). At suture removal, gently tear off the adhesive strips and remove the dressing to expose the wound. The entire process of removing the dressing is easy and does not cause patient discomfort.

## Advantages of the fixation and bandaging method

Simplification of operation: The preoperative hair braiding method solves the problem of difficult hair fixation and easy entry into the surgical field in patients with thick hair, and meanwhile maintains hair stabilization during disinfection and surgery. The postoperative bandaging method cleverly uses the patient's own hair for fixation requiring no additional tools. Technique execution and dressing removal are simple, rapid, and easy to operate.

Even pressure distribution: The dressing applies uniform pressure on the wound, effectively preventing the formation of hematomas or seromas.

Stable fixation: The dressing is securely affixed to the scalp, preventing displacement or detachment.

Protective function: It provides a protective barrier for the wound and prevents contamination.

Patient comfort: The removal of the dressing does not induce pain or discomfort, ensuring a patient-friendly experience.

## Declaration of competing interest

None declared.
